# Estimated Dietary Intakes of Vitamin A5

**DOI:** 10.3390/nu16234004

**Published:** 2024-11-22

**Authors:** Torsten Bohn, Marta Despotovic, Farhad Vahid, Ralph Rühl

**Affiliations:** 1 Nutrition and Health Research Group, Department of Precision Health, Luxembourg Institute of Health, Strassen, L-1445 Luxembourg, Luxembourg; torsten.bohn@lih.lu (T.B.); farhad.vahid@lih.lu (F.V.); 2 Department of Nutrition and Metabolism, Institute for Medical Research, 11000 Belgrade, Serbia; martadespotovic@yahoo.com; 3 CISCAREX UG, Transvaalstr. 27c, D-13351 Berlin, Germany

**Keywords:** carotenoids, 9-*cis*-beta-carotene, dietary intake recommendations, fruit and vegetable consumption, intake stratification, supplementation

## Abstract

Background: A new vitamin concept, termed vitamin A5, an umbrella term for vitamin A derivatives being direct nutritional precursors for 9-*cis*-13,14-dihydroretinoic acid and further induction of RXR-signaling, was recently identified with global importance for mental health and healthy brain and nerve functions. Dietary recommendations in the range of 1.1 (0.5–1.8) mg vitamin A5 / day were suggested by an international expert consortium. The ensuing question arises as to the current daily dietary intake amounts in Western civilization. Methods: Addressing this answer included calculating the intake based on known amounts of vitamin A5 in frequently consumed food items of the human diet that are high in this vitamin, as well as the known daily dietary intake amounts of those selected food components in Westernized countries. Results: Regarding food items, amounts of vitamin A5 in the form of provitamin A5 (i.e., 9-*cis*-beta-carotene (9CBC)), the predominant form in the diet, were found to range from 0.1 to 39 µg 9CBC / g for individual fruits and vegetables, with the highest concentrations being in leafy vegetables. The average intake amounts of vitamin A5 in adults of the general population following a Western lifestyle in Europe averaged 0.9, with a range from 0.5 (for Austria) to 1.3 (for Italy) mg 9CBC/day. Furthermore, based on our calculations, large parts, i.e., approximately two-thirds, of the population are low, even too low (<1.1 mg/day), in daily vitamin A5 intake. Conclusion: In addition to the importance of nudging the population toward a regrettably non-well-accepted higher intake of fruits and vegetables, an additional fortification and supplementation of vitamin A5 could be considered, similar to other micronutrients that are low in a Westernized diet.

## 1. Introduction

Vitamin A5 is a new vitamin concept that was recently proposed [1,2], and it is the first novel identified vitamin in the last 80 years [1,2,3]. It still needs to be added and further transmitted by governmental organizations as a new independent subcategory to the vitamin A group, as suggested previously [1,3,4,5] and based on the WHO [6,7] and IUPAC definitions [8].

The active form of this vitamin is formed out of its precursors, 9-*cis*-beta-carotene (9CBC) as provitamin A5, originating from plant derived food items [1,2], especially from fruits and vegetables; and 9-*cis*-13,14-dihydroretinol as vitamin A5-alcohol, originating from animal sources [1,2]. These two vitamin A5 derivatives are the nutritional relevant precursors of 9-*cis*-13,14-dihydroretinoic acid (9CDHRA) [9,10], the molecule relevant for the endogenous activation of the nuclear hormone receptor, the retinoid X receptors (RXRs) [1,9,10,11].

Vitamin A5 constitutes an umbrella term for all vitamin A derivatives being direct nutritional precursors of 9CDHRA and further RXR–ligand activation, enabling further control and initiation of RXR-mediated signaling [3,4,5,11]. This RXR-mediated signaling is described as being essential for various physiological functions in the mammalian and especially human organism [12,13]. Part of the focus is on the RXR-specific and selective nerve- and brain-relevant physiological processes, such as control of dopamine signaling [14,15], myelination / re-myelination [16], brain clean-up [17], and cholesterol homeostasis / -efflux [18], as recently summarized [4]. These physiological processes are responsible for the maintenance of good mental health, including maintenance of cognition and mental stress-resistance [4], as well as the prevention of major neurological diseases, such as multiple sclerosis, schizophrenia, Alzheimer’s and Parkinson’s disease, and dementia [4].

Various animal-based studies targeting stress / anxiety and depression [19], as well as cognition [1,9], with proven RXR-mediated signaling dependence have thereby clearly been connected with vitamin A5 - RXR-mediated signaling, thereby being directly dependent on vitamin A5 intake and further initiation and control of vitamin A5-dependent activity, and this also applies to humans [20]. Targeted evaluations of already-existing studies from the NUTRITECH EU-FP7 cohort focusing on correlations based on vitamin A5 intake and endogenous levels of vitamin A5 with mental health parameters and brain/nerve-related functional markers are ongoing, and further clinical intervention studies targeting mental health and neurological diseases will soon be launched.

Historically, it was only after clearly observable vitamin deficiencies were identified in humans that the underlying individual and health-relevant mechanism of action was identified. Later on, further detailed dietary recommendations for vitamins were finally suggested by national and international authorities, such as the USDA in the USA [3] or EFSA for Europe [21]. Traditionally, vitamin intake recommendations were based on studies — human observational or even intervention studies or animal ones — that prevented typical, i.e., vitamin-related, deficiency symptoms [3,6]. Today, 80 years after the discovery of the last vitamin [22], different strategies for the identification and establishment of potential novel vitamin concepts are more appropriate. Such a novel strategy is needed, mostly due to ethical restrictions for deprivation and supplemental dose-escalating studies of perceived important dietary constituents in humans [23] and regarding animal studies in sight of difficulties to transfer findings from animal models to humans, due to different metabolism and body weight [23,24]. In addition, strategies performed around 100 years ago included randomly supplementing people with diseases in a non-ethical and non-standardized way and are not possible anymore [25]. Such strategies have been replaced by extremely work-intensive and highly expensive standardized clinical procedures. Targeted supplementation trials that exclude specific food components rich in a specific vitamin in a person’s diets, such as performed earlier for vitamin B3, employing war prisoners [26], are for obvious reasons not ethically possible anymore.

Instead, today, a backward strategy, starting (i) from a unique mechanism of action involving the active vitamin form in blood/tissues and (ii) relevant cellular concentrations related to normal physiological metabolism toward (iii) considering nutritional precursors from food components and calculation of their daily intake amounts, also accounting for (iv) aspects of their bioavailability, is a more feasible and logical strategy [27,28].

In summary, establishing a new vitamin category today requires different strategies and measures than a century ago, especially using State-of-the-Art laboratory experiments for establishing a novel food-dependent mechanism of action with crucial health benefits.

Regarding the large picture for vitamin A5, many of these puzzle pieces have been sorted out [1,2,3,4,5,9,10,11,19], including its biological relevance as the ligand for the RXR nuclear receptor and the presence of precursor molecules in the diet [1,2]. However, some crucial parts along the way of establishing intake recommendations can only be addressed following a logical step-by-step procedure connecting the various aspects of tissue concentrations, bioavailability and bioconversion aspects, and occurrence in food items together [3].

In this article, we add another important piece to the vitamin A5-concept story by estimating daily intake amounts of this vitamin in Westernized countries. Based on these estimations, the nutritional situation of these countries with respect to vitamin A5 supply can be evaluated, and we can further stratify which subgroups, in regard to age, gender, or country, may be potentially low in their daily intake levels of this novel vitamin.

## 2. Materials and Methods

### 2.1. Vitamin A5 / Provitamin A5 Levels in Food Items

First, we summarized, based on published studies and databases [29,30], the vitamin A5 levels in the form of provitamin A5 (9CBC) in food items, focusing on fruits and vegetables as provitamin A5-rich sources that are also frequently consumed. For this purpose, a review of the published peer-reviewed literature was conducted to identify relevant data on vitamin A5 levels in food items [3].

In addition to the EFSA food-consumption database [30], one additional study that received particular attention was the EPIC cohort [29], as fruit and vegetable intake was available as relevant subcategories, i.e., leafy vegetables, cabbages, root vegetables, fruiting vegetables, and onions / garlic, as well as other vegetables, but this was not the case for the EFSA food-consumption database [30]. We combined the averaged data of 9CBC concentrations in vegetable and fruit subgroups from Table 1 to calculate a weighted average of 0.2 µg 9CBC / g fruits and 5.2 µg 9CBC / g vegetables (Table 2).

### 2.2. Consumption of Fruits and Vegetables, as Well as Their Subgroups

In a second step, we summarized, based on European food-consumption data [30], the average amounts of these food items (i.e., fruits and vegetables) consumed by different populations in individual European countries. For this purpose, due to the large number of available data and due to the standardized approach by which the data were obtained, we have focused largely on EFSA’s food-consumption database, which summarizes food-consumption patterns across member states (displayed in Table 3) [30], and the EPIC cohort based database displayed in Table 4 [29].

The EFSA food-consumption database provided detailed data on food intake patterns, including frequency and quantity of fruit and vegetable consumption, across various demographic groups (age and gender based). When several populations or studies were available for a European country, the arithmetic mean per each study was first calculated. Thereafter, the average consumption per country level was calculated, which was then termed “mean of means”.

In addition to food intake data, information was also collected on fruit and vegetable intake recommendations per country, in order to compare estimated intakes with the respective countries’ food based dietary guidelines.

### 2.3. Calculation of Provitamin A5 Intake

As a third step, we further calculated the intake of 9CBC by means of the more detailed EPIC cohort data by combining the average 9CBC contents from Table 2 with the food-intake data from EPIC (Table 4) per country. Furthermore, we calculated, based on these estimations and the previous intake estimates, the amount of provitamin A5 consumed per day based in each European nation.

### 2.4. Statistical Analysis

Descriptive statistics, including the mean and standard deviation (SD), were computed to summarize the average daily intake levels of provitamin A5 across different population groups. Frequency distributions were also examined to identify patterns of vitamin A5 intake within each population.

**Table 1:** Concentration of 9CBC in µg 9CBC / g food matrix.

	9CBC	9CBC
	Dry Weight in µg/g	Fresh Weight in µg/g
**Food item**		
***Fruits***		
Apricot	4.4	0.6
Banana	0.3	0.1
Cherry	-	-
Date	-	-
Grape	-	-
Guava	-	-
Mango	-	-
Nectarine	-	-
Papaya	7.0	1.3
Peach	-	-
Pear	-	-
Prune	-	-
Peach	-	0.3
**Average of all fruits**	**0.9**	**0.2**
***Vegetables***		
Lettuce #	41	2.0
Spinach #		38.6
Cabbage ##	-	-
Kale ##		14.1
Broccoli ##		5.0
Carrot §	57.1	6.5
Cucumber §§	-	-
Eggplant §§	-	-
Pumpkin (1) §§	2.7	0.2
Tomato (1) §§	-	-
Zucchini §§	-	-
Pumpkin (2) §§		2.5
Tomato (2) §§		4.8
Pepper (red) §§	6.0	0.6
Pepper (red hot) §§	9.6	1.0
Onion (green) $	-	-
Parsley $$	111.2	11.6
Dill $$	27.7	4.3
Potato $$		-
Sweet potato (1) $$	65.1	10.2
Sweet corn $$	-	-
Sweet potato (2) $$		1.5
**Average of all vegetables**	**20.1**	**4.7**

*This table is based on the data present in References [31,32] and further calculations performed in [3]. Some data were not measured, as indicated by a blank space, and some data were below the quantification limit and are indicated by a “-” which was set with 0.05 as half of the quantification limit for further calculation. In addition, we categorized vegetable subgroups as follows:* *#—leafy vegetables; ##—cabbages; §—root vegetables; §§—fruiting vegetables; $—onions / garlic; and $$—other vegetables. Numbers 1 [31] and 2 [32] indicate different levels of 9CBC based on different studies.*

## 3. Results

### 3.1. Concentration of Vitamin A5 in the Form of Provitamin A5 in Individual Food Items

A limited number of publications exists reporting concentrations of vitamin A5 / 9CBC, expressed as provitamin A5, i.e., 9CBC, in fruits and vegetables (Table 1). The average concentration of 9CBC in fresh fruits was found to be 0.2 µg 9CBC / g fruits, and the estimated concentration in vegetables was 4.7 µg 9CBC / g vegetables. Meanwhile, a weighted average estimation, considering different intake amounts of individual vegetable subgroups, resulted in an average of 5.2 µg 9CBC / g vegetables, which was used for all further calculations.

**Table 2:** Calculation of averages from subgroup of vegetables.

	9CBC in µg/g (&)
**Average for leafy vegetables (#)**	20.3
**Average for cabbages (##)**	6.4
**Average for root vegetables (§)**	6.5
**Average for fruiting vegetables (§§)**	1.0
**Average for onions / garlic ($)**	0.1
**Average for other vegetables ($$)**	4.6
**Adjusted average of all vegetables**	5.2 *

Calculation of averages from subgroup of vegetables, such as in Table 1, based on different vegetable subgroups given in the EPIC cohort study [29]. The adjusted average * is based on a calculated average of 13% leafy vegetables, 12% cabbages, 13% root vegetables, 42% fruiting vegetables, 7% onion / garlic, and 14% other vegetables listed in the EPIC cohort study [29]. Symbols: &—as fresh weight of the vegetables; #—leafy vegetables; ##—cabbages; §—root vegetables; §§—fruiting vegetables; $—onions / garlic; and $$—other vegetables.

When comparing different subgroups of vegetables, the highest concentrations appeared to be present in leafy vegetables, followed by root vegetables and cabbages (Table 2). It should be noted that fruit juices, for lack of data regarding 9CBC, were not included in this evaluation.

Data regarding vitamin A5 intake in the form of 9CDHROL and 9CDHROL-esters from animal-based products were not reported, as there are currently no data available, and concentrations have been reported to be very low [1].

### 3.2. Summary of Current Intake Recommendations for Fruits and Vegetables

#### 3.2.1. Daily Intake of Fruit and Vegetables in Europe

- A. Based on the EFSA food-consumption database (Table 3)

**Figure 1:**
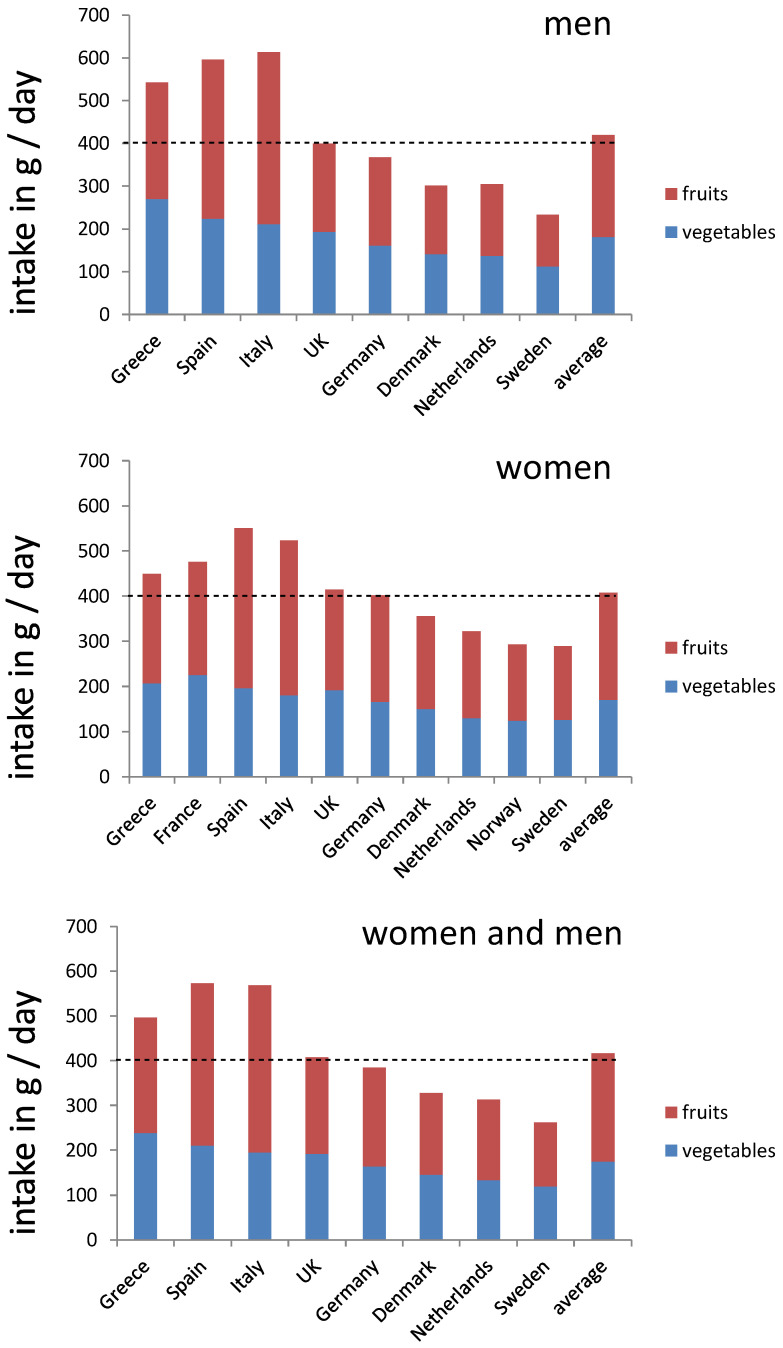
Schematic summary of a dietary pattern focusing on fruit and vegetable intakes in different European countries. The dashed line indicates the recommended “5 A DAY” recommendation of 5 portions of 80 g fruits and / or vegetables for daily intake. The dashed line indicates the recommended intake of 1.1 mg 9CBC (provitamin A5) / day.

**Figure 2:**
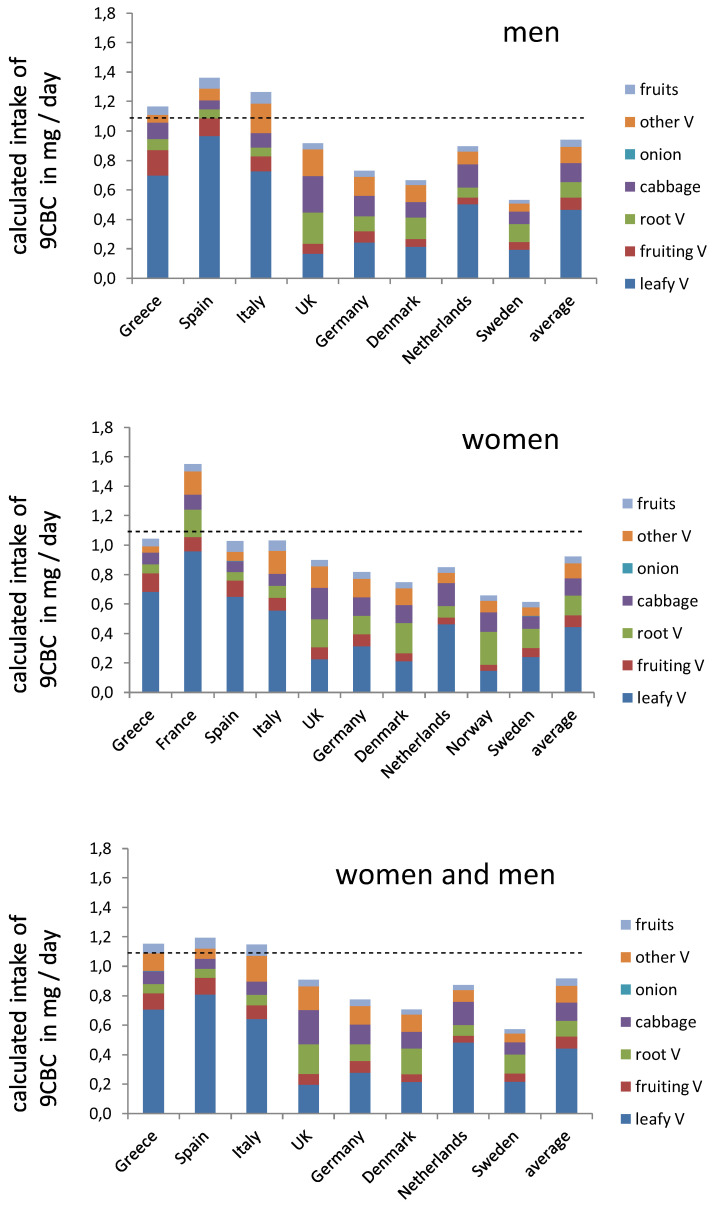
Schematic summary of the calculated vitamin A5 intake levels in different European countries. Used abbreviations; V—vegetables. The dashed line indicates the recommended intake of 1.1 mg 9CBC (provitamin A5) / day.

Data from 24 countries, 23 based on the EFSA food-consumption database, encompassing a total of 26.600 participants aged 18–64 years, were included in this investigation. As shown in Figure 1 and Figure 2 and Table 3, fruit intake ranged from 91 g/day (Sweden and Ireland) to 287 g/day (Luxembourg), with an average of 147 g/day. Vegetable intake was reported to be lowest in Austria (90 g/day) and highest in Italy (236 g/day) and an average of 164 g/day. This translates to the highest combined intake of fruits and vegetables, with 503 g/day in Luxembourg and the lowest in Sweden, with 218 g/day, and an average of 312 g/day.

**Table 3:** Fruits (F) and vegetables (V) intake (g per day) from the EFSA database of different European countries ^Ϯ^.

Countries	Fruits	Vegetables	F + V	% V	Measurement Method and Survey Name	Sample Size	Year
**Italy**	171 ± 143	236 ± 155	407	58	Italian national dietary survey on the adult population from 10 up to 74 years old	726	2018
**Hungary**	138 ± 139	235 ± 166	373	63	Hungarian national food-consumption survey	529	2018
**Serbia**	137 ± 164	217 ± 165	354	62	Serbian Food Consumption Survey on adults	1150	2019
Luxembourg *	287 ± 268	216 ± 172	503	43	ORISCAV-LUX 2 [33]	1326	2017
**Latvia**	141 ± 170	203 ± 133	345	59	Latvian National Dietary Survey	1080	2012
**Cyprus**	111 ± 135	201 ± 145	312	65	National dietary survey of the adult population of Cyprus	272	2014
**Greece**	121 ± 164	192 ± 143	313	61	The EFSA-funded collection of dietary and related data in the general population aged 10–74 years in Greece	260	2014
**Portugal**	161 ± 140	182 ± 130	343	53	National Food, Nutrition, and Physical Activity Survey of the Portuguese general population	3102	2015
**Croatia**	136 ± 153	174 ± 158	310	56	Croatian food-consumption survey on adults	2002	2011
**Estonia**	226 ± 231	168 ± 159	394	43	National Dietary Survey among 11–74-year-old individuals in Estonia	2124	2013
**Montenegro**	120 ± 110	163 ± 121	326	50	Montenegrin National Dietary Survey on the general population	697	2017
**Netherlands**	115 ± 128	163 ± 117	279	59	Dutch National Food Consumption Survey 2012–2016 (DNFCS)	1487	2012
**Romania**	163 ± 186	154 ± 110	317	49	Romanian national food-consumption survey for adolescents, adults, and elderly	740	2019
**Slovenia**	158 ± 151	153 ± 105	311	50	Slovenian national food-consumption survey	385	2017
**Ireland**	91 ± 95	151 ± 84	242	62	North / South Ireland Food Consumption Survey	958	1997
**Bosnia and Herzegovina**	130 ± 144	149 ± 122	279	53	Bosnia-Herzegovinian Dietary Survey of adolescents, adults, and pregnant women	850	2017
**Spain**	155 ± 129	144 ± 102	299	48	Spanish National dietary survey in adults, elderly, and pregnant women	536	2013
**France**	133 ± 131	144 ± 90	277	52	Individual and national study on food consumption 2	2276	2007
**Finland**	171 ± 178	126 ± 102	297	43	National Findiet Surveys	1575	2007
**Sweden**	91 ± 104	126 ± 99	218	59	National Diet and Nutrition Survey—Years 1–3	1266	2008
**Belgium**	118 ± 122	125 ± 100	244	52	Diet National 2004	1292	2004
**Czech Republic**	124 ± 123	117 ± 91	241	49	Czech National Food Consumption Survey	1666	2003
**Germany**	165 ± 175	95 ± 94	260	37	National Nutrition Survey II	10,419	2007
**Austria**	162 ± 153	90 ± 96	252	36	Austrian Study on Nutritional Status 2010–2012-Adults	308	2010
**European average ± STD**	**147 ± 42**	**164 ± 41**	**312 ± 64**	**53 ± 8**	**Calculated sum of the 24 listed surveys**	**37,026**	

*Countries were sorted from top to bottom, based on the average vegetable intake.* Ϯ European food-consumption data [30]. Age ranges from 18 to 64 years. * Median and interquartile range.

**Table 4:** Summarized data from the EPIC cohort based on the data from the year 2002 [29].

Men		Fruits (F)	Vegetables (V)	F + V	Leafy V	% V / (F + V)	% LV / V
Country	n	mean	mean		mean		
**Greece**	1312	273	270	543	30	50	11
**Spain**	1777	372	224	596	44	38	19
**Italy**	1444	403	211	614	35	34	17
**Germany**	2268	207	160	368	14	44	9
**Netherlands**	1024	168	137	305	28	45	20
UK *	518	206	193	400	10	48	5
**Denmark**	1923	160	141	301	10	47	7
**Sweden**	2765	122	112	234	9	48	8
**Sum # / av ##**	**13,031**	**239**	**181**	**420**	**23**	**44**	**12**
STD		102	53	146	13	5	6
							
**Women**							
**Greece**	1374	242	207	449	29	46	14
**Spain**	1443	355	196	551	34	36	17
**Italy**	2512	343	181	524	28	34	16
**Germany**	2150	236	166	403	17	41	10
**Netherlands**	2960	192	130	322	23	40	18
UK *	768	223	192	415	15	46	8
**Denmark**	1995	206	150	356	11	42	7
**Sweden**	3285	164	127	290	11	44	9
France **	4639	251	226	476	45	47	20
Norway **	1798	168	125	293	7	43	5
**Sum # / av ##**	**22,924**	**238**	**170**	**408**	**22**	**42**	**12**
STD		66	36	93	12	4	5
							
**Women and Men**							
**Greece**	2686	257	238	496	29	48	12
**Spain**	3220	363	210	573	39	37	18
**Italy**	3956	373	196	569	32	34	16
UK *	1286	215	193	407	13	47	6
**Germany**	4418	222	163	385	15	42	9
**Denmark**	3918	183	145	328	10	44	7
**Netherlands**	3984	180	133	313	25	43	19
**Sweden**	6050	143	119	262	10	46	8
**Sum # / av ##**	**29,518**	**242**	**175**	**417**	**22**	**43**	**12**
STD		85	41	118	11	5	5

The countries are sorted from top to bottom, based on the vegetable intake of women and men; n—number of included persons; av—average; * only health-aware people were included; ** only women were examined for this country; #—the sum is relevant for the number (n) of included persons; ##—average of fruit intake, vegetable intake, F + V intake, leafy V intake, % V / (F + V), and % LV / V.

Most countries did not even reach the minimum WHO-recommended 400 g of fruits and vegetables / day. According to the EFSA food-consumption database, out of the 23 countries listed in Table 3, only Italy and Luxembourg reached the recommended 400 g of fruits and vegetables / day (i.e., 5 portions per day) target. Especially Southern European countries such as Spain, Greece, Portugal, Italy, and Cyprus showed generally higher combined fruit and vegetable intakes compared to Northern European countries such as Ireland, Denmark, Sweden, or Finland.

- B. The EPIC cohort (Table 4)

When comparing the findings of the EFSA to the EPIC cohort, in which data were generally also obtained by 24 h recalls (though not by multiple ones), similar tendencies were found for fruit and vegetable intakes, with a higher average of 417 g/day in comparison to 312 g/day in the EFSA database. The average vegetable intake in the EPIC database was 175 g/day, and it is comparable to the 164 g/day in the EFSA database. The average fruit intake was 242 g/day in the EPIC cohort, while the EFSA values were much lower, with 147 g/day.

The highest intakes of fruits / vegetables per capita (Table 4) were found comparable to those found in the EFSA database for Southern European nations like Spain (573 g/day), followed by Italy (569 g/day) and Greece (496 g/day), while the lowest ones were mainly determined also comparable in Northern European countries like Sweden (262 g/day) and The Netherlands (313 g/day). With regard to the recommended 400 g of fruit and vegetables per day, only four out of eight countries (Table 4 and Figure 1), for which data were available for both men and women, met this intake recommendation.

#### 3.2.2. Calculation of Individual Intake of Vegetable Subgroups

Regarding vegetable subgroups determined based on the EPIC cohort study, a total of 22 g of leafy vegetables (men and women combined, out of 175 g total vegetables) was consumed, based on data from eight countries reporting the data of both men and women (Table 3).

Regarding fruits, an average of 242 g/day was consumed. Southern countries such as Italy, Spain, and Greece showed consistently higher intakes for vegetables, including leafy ones, and fruits compared to Northern European countries such as Denmark, the Netherlands, Germany, and Sweden. An exception was noted for cabbages, which did not follow this trend, as high concentrations were consumed in the UK, Denmark, and Germany. When looking at subgroups for men or women, no remarkable overall differences were apparent for the intake of total vegetables, leafy vegetables, and individual subgroups (Figure 2).

### 3.3. Estimating the Daily Intake of Provitamin A5

**Figure 3:**
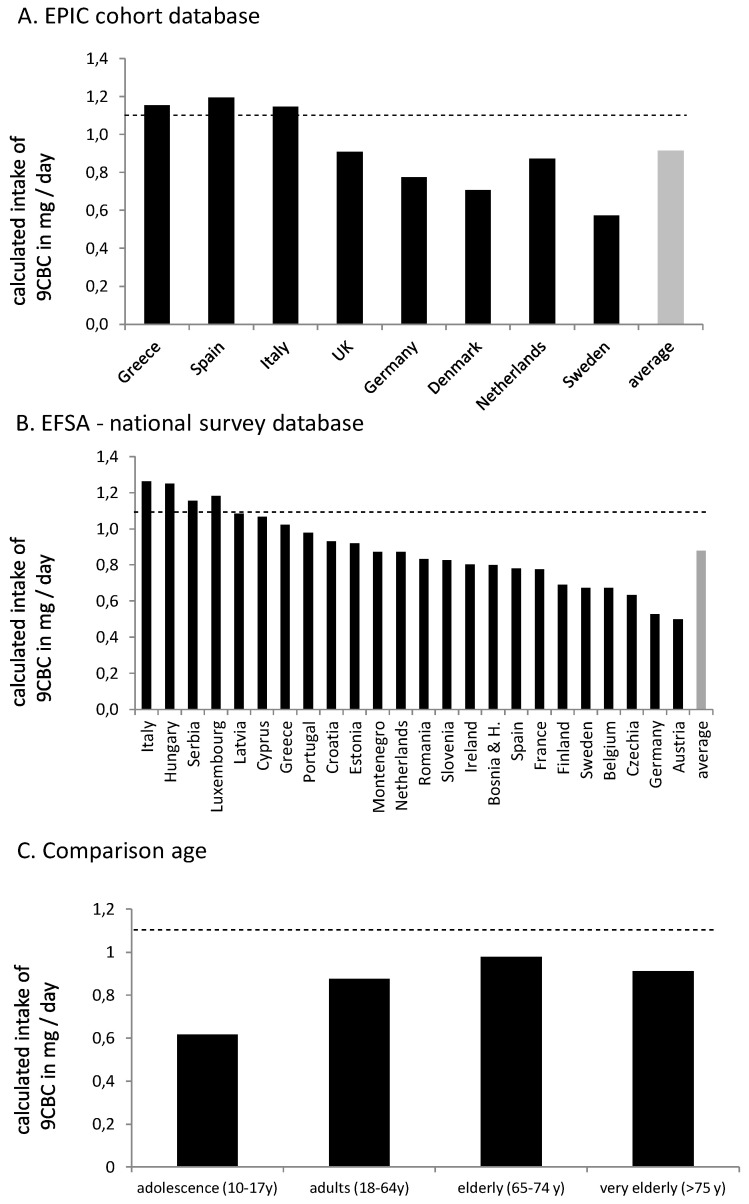
Calculated individual daily 9CBC intake levels based on the data of (**A**) the EPIC cohort [29], (**B**) summarized national surveys, and (**C**) age-dependent intake, based on the EFSA food-consumption database [30]. All estimated values are based on average values for 9CBC in fruits (0.2 µg/g) and vegetables (5.2 µg/g). The dashed line indicates the recommended intake of 1.1 mg 9CBC (provitamin A5) / day.

Based on the EPIC cohort, more than half of the countries would fall below the earlier proposed 1.1 mg/day intake of provitamin A5 (Figure 3). Only the Southern European countries, due to their generally higher intake of fruits and vegetables (Table 3 and Table 4 and Figure 1 and Figure 2) were estimated to reach the 1.1 mg/day target, while Northern and Central European countries had intakes as low as 0.5 mg/day. Employing data from EFASs food-consumption database, and also assuming an approx. content of 9CBC of 0.2 µg/g for fruits and 5.2 µg/g for vegetables (based on Table 2) based on this cruder estimate, only few a countries, like Italy, Hungary, Luxembourg, and Serbia, reached the 1.1 mg/day 9CBC target, while the majority of 19 countries did not reach the suggested 1.1 mg 9CBC intake / day. The European average was comparable with 0.9 mg 9CBC / day in the EPIC cohort and 0.9 mg 9CBC in the EFSA cohort (Figure 3). 

Due to their high content of 9CBC combined with their relatively frequent consumption, leafy vegetables were the vegetable subgroup most largely contributing to total provitamin A5 intake (contributing to often >50% of total 9CBC intake), followed by root vegetables, cabbages, and other vegetables (Figure 2). Fruits contributed, due to their low content of 9CBC, very little to the overall intake, below 5% (Figure 2). Slight differences between men and women were noted, with women showing slightly lower average intakes of 9CBC.

When looking at 9CBC intake by age in the EFSA cohort (Figure 3C), the differences between the age groups appeared to be small, with the highest intakes in elderly adults aged 65–74 years. This is somewhat confirmed by EFSA data [30], as fruit consumption and especially vegetable consumption were also highest for the elderly compared to adolescents and adults.

### 3.4. Comparison of Provitamin A5 Intake in Persons Consuming Low, Medium, and High Amounts of Fruits and Vegetables (Figure 4)

Based on data from a study just performed in France [34], the majority (>50%) consumed less than 3.5 portions fruits and vegetables / day, with an average of 2 portions, reaching, in consequence, an average intake of only 0.6 mg provitamin A5 / day. Furthermore, only 21% of the French population consumed between 3.5 and 5 portions of fruits and vegetables per day, based on an average of 4.2 portions of fruits and vegetables / day and, consequently, an estimated provitamin A5 intake of 1.3 mg 9CBC / day [35]. Only 25% of the French population consumed sufficient fruits and vegetables with five and more portions / day and an estimated provitamin A5 intake of 2.1 mg 9CBC / day [35,36]. In consequence, this means that ~64% (~2/3rd) of the French population consumes too few fruits and vegetables per day, and in consequence, they also have a too-low daily intake of provitamin A5.

**Figure 4:**
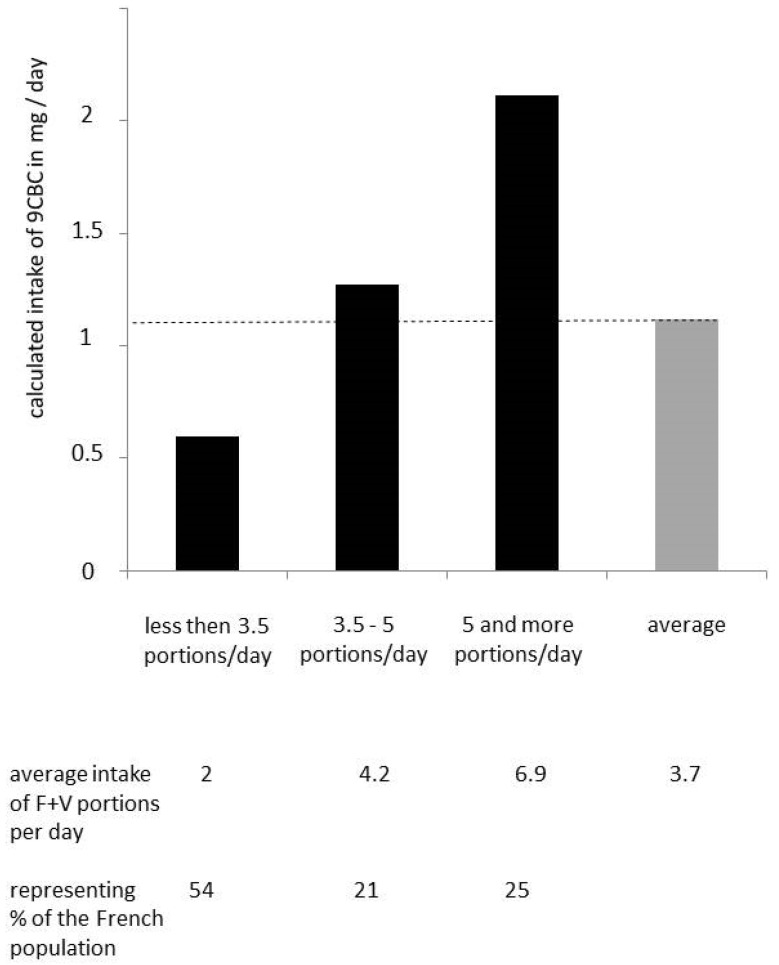
Calculated daily 9CBC intake levels of individuals with low, medium, and high daily average fruit and vegetable intake from a French survey [34] with an estimated average F + V intake of 295 g (F + V) / day. The dashed line indicates the recommended intake of 1.1 mg 9CBC (provitamin A5) / day [3].

## 4. Discussion

In this article we present, for the first time, an estimation of vitamin A5 intake in various European countries. This compound has been identified and categorized as a novel vitamin and / or an independent subcategory of vitamin A, and an intake estimate across various European populations was required. Toward this end, we calculated vitamin A5 intakes independently from vitamin A [1] from the background diet. An average intake of vitamin A5 in the adult population ranging from 0.5 to 1.3 mg per day was found, with a tendency for higher intakes in Southern European countries. It was also estimated that approx. two-thirds of the population would consume less than the proposed recommended intake of 1.1 mg provitamin A5 / day.

Comparable to other vitamins, we make use of available data from cohort studies, in our case, from the EPIC [29] and EFSA cohorts [30], allowing us to calculate the daily intake specified for several European countries, sex, and partly age groups, which are currently available in these databases. As a first step, based on the available data, we calculated the average intake of vitamin A5 in food categories that are rich in vitamin A5. Here, we focused on fruits and vegetables, where provitamin A5, i.e., 9-*cis*-β-carotene, is mainly present. The levels of 9-*cis*-13,14-dihydroretinol, as well as its esters, in the human food matrix have, thus far, either not been determined or are deemed very low [1,2], suggesting that the main intake of vitamin A5 is via plant foods rich in provitamin A5 [3,4]. At first, an average provitamin A5 concentration was calculated, with 0.2 µg provitamin A5 / day for fruits and 5.2 µg provitamin A5 / day for vegetables, which were then employed for further calculations. This was required, as most databases do not contain further details on the consumption of individual fruit and vegetable subgroups.

The highest daily intake amounts of fruits and vegetables were determined in Southern European countries such as Greece, Spain, and Italy; and the lowest were found in Northern European countries, including Sweden, the Netherlands, and Denmark. Remarkable is that the large majority of Europeans was consuming less than the suggested “5 A DAY”-intake recommendations of five portions of each 80 g fruits and vegetables recommended by national and international food organizations [29,30,37]. However, this has been remarked in earlier consumption surveys in Europe [29,34,38,39].

Regarding the best sources of provitamin A5 per weight, the main source of provitamin A5 intake originates from the vegetable subgroup leafy vegetables, with 20.3 µg provitamin A5 / g food matrix, followed by cabbages (6.4 µg/g) and root vegetables (6.4 µg/g), with low concentrations in fruiting vegetables (1.0 µg/g) and onions / garlic (0.1 µg/g). The European country-specific daily intake amount of these leafy vegetables was highest in Southern European countries such as Greece, Spain, and Italy and lowest in Northern / Central European countries, including UK, Norway, Denmark, and Sweden. This is in line with studies reporting higher fruit and vegetable intake in general in Southern European countries [29,40].

Based on the fruit and vegetable intake amounts from the EPIC and the EFSA database [29,30], summarizing 65,544 individuals, we calculated for 27 European countries country-specific daily vitamin A5 intake amounts, with an average of 0.9 mg provitamin A5 / day, which is lower than the previously suggested recommendation of 1.1 mg provitamin A5 / day, calculated by two independent methods. These estimated average intake amounts of provitamin A5 on average of European countries sound moderate / fair compared to the suggested intake amounts. When stratifying for different countries, we observed that the average amounts in the non-Southern European countries, especially Austria (0.5 mg/day), Germany (0.5 mg/day), the Czech Republic (0.6 mg/day), and Sweden (0.7 mg/day EFSA data / 0.6 mg/day EPIC data), were clearly below the suggested intake amounts of 1.1 mg provitamin A5 / day.

Based on the estimations of daily provitamin A5 intake from the 27 European countries, only 4 out of 24 countries for the EFSA database calculations and 3 out of 8 from the EPIC database calculations, in total 6 out of 27 countries, reach sufficient high daily provitamin A5 intake levels on average, while a majority of the European countries are below 1.1 mg provitamin A5 / day. In addition, 3 (based on the EPIC database) and 8 (based on the EFSA database) countries, and in summary, 9 out of 27 countries in total, are even below 0.8 mg provitamin A5 / day on average and thereby far below the suggested daily intake amounts of provitamin A5. This means that a large majority of European countries, on average, have a too-low daily intake of vitamin A5. However, it also needs to be specified that both the intake recommendation and the calculated intake are based on average values for adults, and there are a limited amount of data available. Further studies should aim to determine detailed estimations, such as estimating an intake that would cover over 97% of the population’s need, such as necessary to determine the population reference intake (PRI).

Furthermore, based on evaluations in France [34], it was calculated that 54% of the French population consumes less than 3.5 daily portions of fruits and vegetables, and a further considerable fraction (21%) consumes between 3.5 and 5 portions, meaning that ca. 64% of the French population is below the suggested “5 A DAY” recommendations. This means, as a consequence, that approximately two-thirds of the French population, as well as transferable to many other European populations, are below the suggested daily intake levels of vitamin A5 which were based on the “5 A DAY” concept.

## 5. Summary

Due to the low intake of fruits and vegetables [37], especially vegetables [29,41,42], and with a special focus on low intake of leafy vegetables [29,41,42], there is an estimated low, even too low, daily dietary intake of vitamin A5 present in Europeans. These data are likely relevant for other rather Western lifestyle-based societies following Western-based dietary patterns, including large populations in many developing countries [3,43]. We recommend, based on these calculated parameters, that, in order to improve dietary patterns and assure also sufficient intake of vitamin A5, (a) novel dietary recommendations should focus mainly on a higher daily dietary intake of vegetables and especially leafy vegetables and (b) consider additional food fortification with vitamin A5 and / or dietary supplements to meet the suggested daily dietary intake recommendations for vitamin A5 to avoid a potential vitamin A5 deficiency [4].

Whether a low daily intake of vitamin A5 is associated with mental problems, which are prevalent in our Western hemisphere [4], is currently merely predicted and requires further attention based on European-wide epidemiological databases with sufficient details for specific European countries regarding dietary intake and mental health issues. Not surprisingly, in Southern European countries such as Greece, Italy, and Croatia, with high average estimated vitamin A5 intakes, the mental problems appear to have a lower prevalence, while in Northern European countries including the Netherlands, Finland, Denmark, Sweden, and Germany, these mental problems are reported to have a high prevalence [44]. A direct correlation and causal connection must still be elaborated and evaluated in larger databases, together with deeper functional mechanistic evaluations. Furthermore, described and suggested long-term or medium-term vitamin A5 deficiencies related to issues such as poor mental health and a high incidence of numerous neurological diseases, should be evaluated with respect to nutritional or functional vitamin A5-signaling pathways [4]. Poor mental health is described to be based on a low daily intake of fruits and vegetables, especially vegetables [45], with a special focus on leafy vegetables [41]. A detailed clinical mechanistic step-by-step approach to how fruits and vegetables, with a focus on vegetables and leafy vegetables rich in vitamin A5, transmit their health-promoting and -protecting activity via the retinoid X receptor was summarized in a recent article [3,4], which is based on dozens of mechanistic studies performed using laboratory models as a base.

In this study, a more detailed and data-based intake of vitamin A5 in several European countries was attempted, and the results were compared against a first recommendation for vitamin A5 intake. The calculated data were evaluated in an ideal-case dietary scenario, as well as in a reality-based scenario, showing that large parts of the European society may have too low of a daily intake of vitamin A5.

This investigation also has some limitations, as the available food-intake data that originated from the EPIC [14] and EFSA databases [30] represent merely average intakes on a population level, and no individual intakes are available. Therefore, no further correlation analyses could be carried out. In addition, there are no individual health-related data available, especially concerning mental and neurological health status in the published EPIC and EFSA data [29,30]. Furthermore, the number of data regarding 9CBC in fruits and vegetables available is still limited, and the data need to be extended further for more precise estimates of dietary intake of vitamin A5.

## 6. Conclusions

Based on the importance of vitamin A5 for the maintenance of good mental health, as well as for the prevention of major neurological diseases, we estimated that the vitamin A5 intake of about two-thirds of Europeans, with a focus on the younger generation (<18 years) and Northern and Central Europeans, is currently below the suggested vitamin A5 intake levels of 1.1 mg provitamin A5 / day. This may be associated with non-optimal protection for general brain and nerve health.

We further recommend a general increase in daily vitamin A5 intake, preferably via a balanced and healthy dietary pattern rich in vegetables, or alternatively, via food fortification / dietary supplementation, such as relevant for other vitamins, in order to assure sufficient vitamin A5 intake for optimal maintenance of good mental health and reducing the high prevalence of major neurological diseases, focusing on countries with a predominant Westernized lifestyle.

These dietary recommendations for vitamin A5 need to be swiftly adapted and further transmitted to the general public and to relevant health and food experts via major health / food organizations, including international organizations such as the WHO and EFSA, as well as numerous national organizations.

## Data Availability

The original contributions presented in this study are included in the article. Further inquiries can be directed to the corresponding author. The data are not publicly available due to legal reasons.
